# From AI literacy to AI-integrated inquiry-based science teaching: the serial mediating roles of AI-TPACK and science teaching self-efficacy among Chinese pre-service science teachers

**DOI:** 10.3389/fpsyg.2026.1911909

**Published:** 2026-09-07

**Authors:** Jun Zou, Nan Li, Xiaofei Wang, Juan Du

**Affiliations:** 1 Department of Chinese Language Studies, Faculty of Humanities, The Education University of Hong Kong, Hong Kong SAR, China; 2 Tianjin Foreign Languages School, Tianjin, China; 3 School of Computer Science and Informatics, University of Liverpool, Liverpool, United Kingdom; 4 School of Information Technology, Monash University Malaysia, Bandar Sunway, Malaysia

**Keywords:** AI literacy, AI-TPACK, inquiry-based science teaching, pre-service science teachers, science teaching self-efficacy

## Abstract

**Introduction:**

Artificial intelligence (AI) is increasingly being integrated into science education, creating new opportunities for visualization, simulation, data interpretation, inquiry-based learning, and scientific explanation. However, it remains unclear how pre-service science teachers translate AI literacy into the professional knowledge and confidence required for AI-supported inquiry-based science teaching. Drawing on AI-TPACK and teacher self-efficacy perspectives, this study examined the serial mediating roles of AI-TPACK and science teaching self-efficacy in the association between AI literacy and Chinese pre-service science teachers’ intention to integrate AI into inquiry-based science teaching.

**Methods:**

A total of 548 Chinese pre-service science teachers from universities participated in the survey. Data were analyzed using partial least squares structural equation modeling.

**Results:**

AI literacy was positively associated with AI-TPACK, AI-TPACK was positively associated with science teaching self-efficacy, and science teaching self-efficacy was positively associated with AI integration intention. Further analysis supported a serial indirect pathway linking AI literacy to AI integration intention through AI-TPACK and science teaching self-efficacy.

**Discussion:**

The findings suggest that AI literacy does not automatically translate into an intention to integrate AI into teaching; rather, this association operates through contextualized technological, pedagogical, and content knowledge and positive capability beliefs. This study extends the application of the AI-TPACK framework to pre-service science teacher education and offers practical implications for systematically developing pre-service science teachers’ AI-related pedagogical competence and integration confidence.

## 1 Introduction

The rapid development of generative artificial intelligence (GenAI) and other AI technologies has created new opportunities and challenges for science education. Recent research indicates that GenAI can support science lesson planning, scientific explanation, inquiry design, and the generation of instructional resources for science learning (Cooper, 2023). These affordances are particularly relevant to science education because AI-based tools can assist pre-service teachers in developing visualizations, simulations, explanations, and inquiry-oriented learning activities (Lee and Zhai, 2024). However, concerns have also been raised regarding the accuracy of AI-generated content, students’ overreliance on GenAI, and the lack of structured guidance in teacher education for the critical use of GenAI (Lee and Zhai, 2024; Spasopoulos et al., 2025). Integrating AI into science teacher education should therefore not be treated merely as a matter of tool use. Instead, pre-service science teachers need the literacy, pedagogical knowledge, and confidence required to evaluate AI-generated scientific content and translate AI’s affordances into meaningful inquiry-based science teaching.

This issue is particularly important in the Chinese context. In recent years, China has strengthened policy support for science education and AI-enabled educational reform. In 2025, the General Office of the Ministry of Education issued national guidance on strengthening science education in primary and secondary schools, emphasizing the development of a more comprehensive science curriculum, a stronger science teaching workforce, and better integration of science education resources (General Office of the Ministry of Education of the People’s Republic of China, 2025). In 2026, five national agencies jointly issued the “Artificial Intelligence + Education” Action Plan, which aims to establish an AI literacy system spanning all levels of schooling and lifelong learning while integrating AI education into local curricula, interdisciplinary teaching, after-school services, and higher education programs (Ministry of Education of the People’s Republic of China et al., 2026). These policy developments indicate that future science teachers in China will increasingly be expected to integrate AI-supported tools into science learning, inquiry activities, and interdisciplinary problem solving. Nevertheless, empirical evidence remains limited regarding how Chinese pre-service science teachers develop the competence and confidence required to integrate AI into inquiry-based science teaching.

Recent empirical studies have begun to investigate pre-service science teachers’ readiness and intention to use AI in science education. Ramnarain et al. (2025) examined pre-service science teachers’ intention to use generative AI in inquiry-based teaching and found that AI literacy, subjective norms, attitudes toward AI, perceived behavioral control, and perceived usefulness significantly predicted intention, whereas perceived skill readiness and concerns about generative AI did not. Using structural equation modeling, Amandoron et al. (2025) also examined pre-service science teachers’ preparedness to integrate AI into science teaching and found that prior technological experience, attitudes toward AI, confidence in learning AI, and engagement in AI learning were associated with AI readiness and preparedness. These studies provide valuable evidence concerning predictors of AI readiness and intention, but they primarily focus on identifying influential factors. Less is known about the professional learning mechanism through which AI literacy is associated with subject-specific AI-related pedagogical knowledge, science teaching self-efficacy, and intention to integrate AI into inquiry-based science teaching.

Another emerging body of research focuses on AI-TPACK, which extends technological pedagogical content knowledge to AI-integrated teaching. Çelik (2023) proposed the intelligent-TPACK framework to explain the professional knowledge teachers require to integrate AI-based tools into education ethically. In science teacher education, Antonio (2025) designed an AI-focused pedagogical learning course for pre-service science teachers and reported significant improvements across AI-TPACK dimensions, including technological knowledge, pedagogical application, ethical considerations, and integrated competence. This evidence indicates that AI-TPACK can be strengthened through deliberately designed teacher education interventions. However, this line of research has mainly examined whether AI-TPACK can be enhanced through coursework rather than whether it functions as a mechanism linking AI literacy with science teaching self-efficacy and future intention to integrate AI into teaching.

Science teaching self-efficacy may constitute a key psychological mechanism in this process. Recent science teacher education research indicates that technology-enhanced, student-centered, and practice-based learning experiences can strengthen pre-service teachers’ science teaching self-efficacy (Ingram et al., 2024; Ribeirinha and Correia, 2025). For example, Ingram et al. (2024) found that garden-based technology integration and practicum implementation enhanced elementary pre-service teachers’ personal science teaching efficacy. Ribeirinha and Correia (2025) similarly reported that a flipped classroom model strengthened pre-service teachers’ science teaching self-efficacy beliefs, particularly personal science teaching efficacy. These findings suggest that efficacy beliefs can be strengthened through structured pedagogical experiences. Nevertheless, the mediating role of science teaching self-efficacy remains underexplored in the context of AI-integrated science teaching. This gap is important because pre-service teachers may possess AI-related knowledge while still lacking confidence in using AI to support inquiry, experimentation, data interpretation, and scientific explanation.

To address these gaps, the present study examined how AI literacy statistically predicts Chinese pre-service science teachers’ intention to integrate AI into inquiry-based science teaching. Specifically, it investigated the serial mediating roles of AI-TPACK and science teaching self-efficacy. AI literacy was defined as pre-service science teachers’ ability to understand, use, evaluate, and ethically apply AI tools in science teaching contexts. AI-TPACK referred to their perceived ability to integrate AI tools with science content and inquiry-based pedagogy. Science teaching self-efficacy referred to their confidence in effectively teaching science and supporting students’ science learning. Intention to integrate AI into inquiry-based science teaching referred to their willingness and plans to use AI tools in future science classrooms to support inquiry questions, scientific modeling, simulated experiments, experimental work, data interpretation, and explanation construction. The study addressed the following research questions:

RQ1: Does AI literacy statistically predict Chinese pre-service science teachers’ intention to integrate AI into inquiry-based science teaching?

RQ2: Does AI-TPACK mediate the association between AI literacy and intention to integrate AI into inquiry-based science teaching?

RQ3: Do AI-TPACK and science teaching self-efficacy serially mediate the association between AI literacy and intention to integrate AI into inquiry-based science teaching?

## 2 Literature review and hypotheses

### 2.1 AI literacy in pre-service science teacher education

AI literacy refers to pre-service science teachers’ ability to understand, evaluate, ethically use, and apply AI tools capable of generating or processing text, images, graphs, simulations, data, audio, and video in science teaching contexts (Pingmuang et al., 2025; Spasopoulos et al., 2025). Such literacy is especially important in science education because GenAI can support lesson design, scientific explanations, visualization, inquiry activities, and instructional resource generation. It also requires teachers to critically evaluate the scientific accuracy and pedagogical appropriateness of AI-generated content (Cooper, 2023). Pingmuang et al. (2025) developed and validated an AI literacy scale for pre-service teachers comprising four dimensions: AI recognition, fundamental AI comprehension, AI pedagogy, and ethical AI use. This framework indicates that AI literacy in teacher education should encompass both pedagogical and ethical competence. Although AI literacy provides a foundation for teachers’ use of AI, it is not equivalent to subject-specific AI teaching competence. Maurer et al. (2026) found no significant association between the general AI literacy of the surveyed science educators and their actual use of AI, suggesting that general AI knowledge does not necessarily translate into meaningful applications in science teaching. Science teachers additionally need to understand subject-specific applications of AI in scientific data processing, simulation and modeling, evidence evaluation, and scientific explanation (Huwer et al., 2025). Although the AI literacy measure used in this study was contextualized for science teaching, it primarily assessed AI recognition, fundamental comprehension, pedagogical application, and ethical use rather than direct performance in these disciplinary practices. Accordingly, this study conceptualized AI literacy as a foundational construct and AI-TPACK as a professional knowledge mechanism linking general AI knowledge with science teaching integration. Pre-service science teachers with stronger AI literacy may therefore be more likely to recognize the pedagogical value of AI for visualizing scientific concepts, simulating experiments, interpreting data, constructing scientific models, designing inquiry questions, and diagnosing students’ misconceptions.

*H1*: AI literacy positively predicts AI-TPACK among Chinese pre-service science teachers.

### 2.2 AI-TPACK: professional knowledge for AI-integrated science teaching

AI-TPACK extends the traditional TPACK framework by emphasizing that teachers need to understand how AI tools can be integrated with subject matter, pedagogy, and ethical decision making (Çelik, 2023; Mishra et al., 2023). This perspective is particularly relevant to science teaching because science teachers must determine whether AI-generated explanations, graphs, simulations, models, and data analyses are scientifically accurate and pedagogically appropriate (Lee and Zhai, 2024; Spasopoulos et al., 2025). Antonio (2025) found that an AI-focused pedagogical learning course significantly enhanced pre-service science teachers’ AI-TPACK, including technological knowledge, pedagogical applications, ethical considerations, and competence in AI-integrated teaching. However, AI-TPACK is not the only framework for explaining teachers’ professional competence in the AI era. The DPACK model proposed by Thyssen et al. (2023) extends TPACK by incorporating the sociocultural dimension of digitality. In natural science education, the DiKoLAN AI framework developed by Huwer et al. (2025) further highlights the subject-specific nature of AI-related competence. In light of these theoretical developments, the present study adopted AI-TPACK as the central construct in the structural model but did not treat it as a comprehensive measure of pre-service science teachers’ AI competence. TPACK conceptualizes teachers’ technology integration knowledge through the dynamic interaction among technological, pedagogical, and content knowledge (Koehler et al., 2013). In this study, AI-TPACK specifically referred to pre-service science teachers’ perceived ability to integrate AI tools, science content, inquiry-based pedagogy, and ethical judgment. This construct was therefore appropriate for examining associations among AI literacy, professional knowledge, self-efficacy, and AI integration intention. Pre-service teachers with stronger AI literacy may consequently report stronger AI-TPACK because they are better able to understand the functions, limitations, and ethical risks of AI in science teaching.

*H2*: AI-TPACK positively predicts science teaching self-efficacy.

### 2.3 Science teaching self-efficacy in AI-supported inquiry-based teaching

Science teaching self-efficacy refers to pre-service teachers’ beliefs in their ability to teach scientific concepts, organize science learning activities, support students’ scientific understanding, and manage inquiry-based learning processes (Ingram et al., 2024; Ribeirinha and Correia, 2025). Recent studies indicate that structured, technology-enhanced, and student-centered learning experiences can strengthen pre-service teachers’ personal science teaching efficacy (Ingram et al., 2024; Ribeirinha and Correia, 2025). In AI-supported inquiry-based teaching, AI-TPACK may be positively associated with science teaching self-efficacy because understanding how to align AI tools with science content and pedagogy may reduce uncertainty in instructional design (Antonio, 2025; Lee and Zhai, 2024). Pre-service teachers who feel more confident in science teaching may also be more willing to use AI in future classrooms to support inquiry, experimentation, data interpretation, and scientific explanation.

*H3*: Science teaching self-efficacy positively predicts intention to integrate AI into inquiry-based science teaching.

### 2.4 Serial mediation model

Intention to integrate AI into inquiry-based science teaching refers to the extent to which pre-service science teachers are willing and plan to use AI tools in the future to support inquiry-question design, experimental work, evidence collection, data interpretation, scientific modeling, and the construction of scientific explanations (Ramnarain et al., 2025). Ramnarain et al. (2025) found that AI literacy, subjective norms, attitudes toward AI, perceived behavioral control, and perceived usefulness significantly predicted pre-service science teachers’ intention to use generative AI in inquiry-based teaching. Amandoron et al. (2025) further found that prior technological experience, attitudes toward AI, confidence in learning AI, and engagement in AI learning were associated with pre-service science teachers’ AI readiness and preparedness for AI integration. Nevertheless, these studies primarily examined predictors of AI readiness or intention and paid less attention to the mechanisms through which AI literacy may be associated with AI-TPACK, science teaching self-efficacy, and AI integration intention.

*H4*: AI-TPACK mediates the association between AI literacy and intention to integrate AI into inquiry-based science teaching.

The present study proposed that AI literacy first supports AI-TPACK because pre-service teachers need to understand and evaluate AI tools before integrating them with science content and inquiry-based pedagogy (Çelik, 2023; Pingmuang et al., 2025). AI-TPACK may subsequently strengthen science teaching self-efficacy because teachers who know how to integrate AI, science content, and pedagogy may be more likely to believe that they can design and implement AI-supported science lessons (Antonio, 2025; Ingram et al., 2024). Science teaching self-efficacy may, in turn, strengthen AI integration intention because efficacy beliefs are closely related to pre-service teachers’ confidence in implementing science teaching practices and technology-supported instruction (Ingram et al., 2024; Ribeirinha and Correia, 2025). This proposed sequence addresses a gap in the literature. Although recent studies have separately examined AI literacy, AI readiness, AI-TPACK, and AI use intention, few have tested how these constructs operate jointly in AI-integrated science teacher education (Amandoron et al., 2025; Antonio, 2025; Ramnarain et al., 2025).

*H5*: Science teaching self-efficacy mediates the association between AI-TPACK and intention to integrate AI into inquiry-based science teaching.

*H6*: AI-TPACK and science teaching self-efficacy serially mediate the association between AI literacy and intention to integrate AI into inquiry-based science teaching.

### 2.5 Conceptual framework

**Figure 1:**
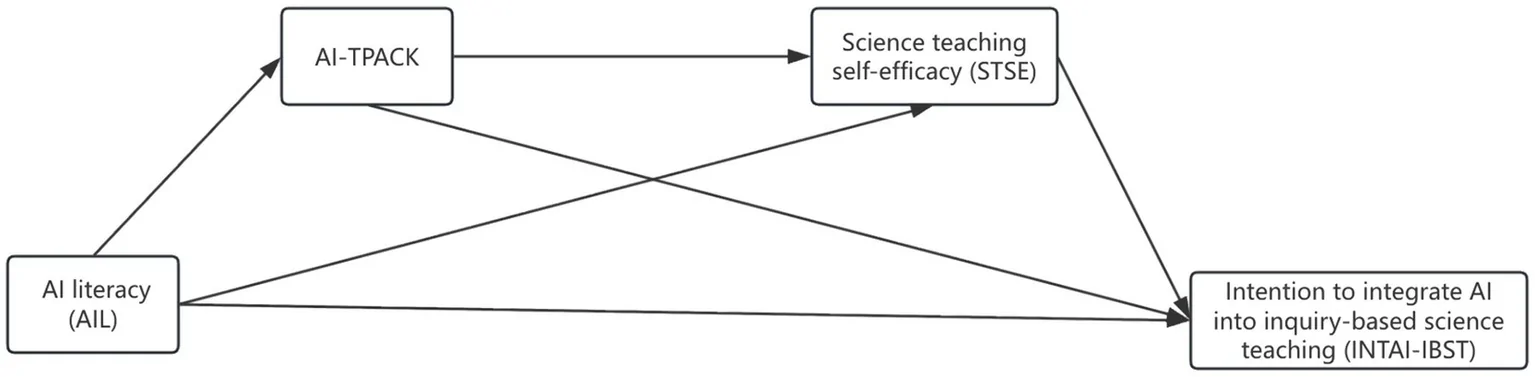
Conceptual framework.

As shown in Figure 1, AI literacy (AIL) was specified as the independent variable, and intention to integrate AI into inquiry-based science teaching (INTAI-IBST) was specified as the dependent variable. AI-TPACK and science teaching self-efficacy (STSE) were modeled as serial mediators to examine the mechanism underlying Chinese pre-service science teachers’ intention to integrate AI into teaching.

## 3 Methods

### 3.1 Participants and procedure

The participants were 548 Chinese pre-service science teachers enrolled in teacher education programs at normal universities or comprehensive universities in China. Their majors included science education, physics education, chemistry education, biology education, geography education, and related science teacher education fields. Most participants were second-, third-, or fourth-year undergraduates who had generally completed courses in educational technology, science teaching methods, or subject-specific pedagogy and therefore possessed a basic foundation in science teaching and instructional design. This population was selected to ensure that participants could make reasonably informed judgments about AI-supported science teaching and inquiry-based instructional design.

This study used convenience sampling to collect data through the Wenjuanxing platform. Before completing the questionnaire, participants were informed of the study purpose, the voluntary nature of participation, the anonymity of the survey, and the exclusive use of the data for academic research. Participants entered the questionnaire only after providing consent. A total of 550 questionnaires were received. Data screening excluded two responses: one showing an obvious invariant response pattern, with the same option selected for every item, and one duplicate submission. The final analytical sample therefore comprised 548 valid questionnaires. The questionnaire consisted of two sections. The first collected demographic information, including gender, age, year of study, major, university type, teaching practicum experience, prior educational technology or science teaching methods coursework, frequency of AI tool use, and AI-related training experience. The second measured the four focal constructs: AI literacy, AI-TPACK, science teaching self-efficacy, and intention to integrate AI into inquiry-based science teaching.

As shown in Table 1, the final sample comprised 548 Chinese pre-service science teachers, including 176 men and 372 women. In terms of age, 168 participants were aged 18–20 years, 291 were aged 21–22 years, and 89 were aged 23 years or older. The sample included 151 s-year, 226 third-year, and 171 fourth-year students. Participants majored in science education, physics education, chemistry education, biology education, or geography education. In addition, 356 participants attended normal universities and 192 attended comprehensive universities. Regarding prior experience, 241 participants had completed a teaching practicum, and 427 had taken educational technology or science teaching methods coursework. Most participants reported using AI tools sometimes or often, and 219 had received AI-related training.

**Table 1:** Demographic characteristics of the participants.

Variable	Category	Frequency	Percentage
Gender	Male	176	32.10%
	Female	372	67.90%
Age	18–20	168	30.70%
	21–22	291	53.10%
	23 years or older	89	16.20%
Year of study	Second year	151	27.60%
	Third year	226	41.20%
	Fourth year	171	31.20%
Major	Science education	116	21.20%
	Physics education	104	19.00%
	Chemistry education	112	20.40%
	Biology education	128	23.40%
	Geography education	88	16.10%
University type	Normal university	356	65.00%
	Comprehensive university	192	35.00%
Teaching practicum experience	Yes	241	44.00%
	No	307	56.00%
Educational technology/science teaching methods coursework	Yes	427	77.90%
	No	121	22.10%
AI use frequency	Rarely	96	17.50%
	Sometimes	213	38.90%
	Often	171	31.20%
	Very often	68	12.40%
AI-related training experience	Yes	219	40.00%
	No	329	60.00%

### 3.2 Measures

All items were measured on a seven-point Likert scale ranging from 1 (strongly disagree) to 7 (strongly agree), consistent with the self-report Likert-type measures commonly used in research on AI literacy, AI-TPACK, teacher self-efficacy, and AI integration intention.

#### 3.2.1 AI literacy

The AI literacy scale was adapted from the pre-service teacher AI literacy scale developed by Pingmuang et al. (2025), which comprises four dimensions: AI recognition, fundamental AI comprehension, AI pedagogy, and ethical AI use. The items were revised for the specific contexts of science teaching and AI tools, with emphasis on AI-supported visualization, simulation, scientific explanation, inquiry-activity design, and ethical evaluation (Lee and Zhai, 2024; Spasopoulos et al., 2025). Seven adapted items were used to assess pre-service teachers’ perceived ability to understand, use, evaluate, and ethically apply AI tools in science teaching contexts.

#### 3.2.2 Ai-TPACK

The AI-TPACK scale was adapted from the AI-TPACK Survey for Pre-Service Teachers used by Antonio (2025) in a study of pre-service science teachers. The original instrument assessed AI-related technological knowledge, pedagogical application, ethical considerations, and competence in AI-integrated teaching. Because this study specified AI-TPACK as a first-order construct within a serial mediation model, it was operationalized as pre-service science teachers’ perceived ability to integrate AI tools, science content, and inquiry-based pedagogy (Antonio, 2025; Çelik, 2023). Ten adapted items were used, all revised to fit the science teaching context.

#### 3.2.3 Science teaching self-efficacy

The science teaching self-efficacy scale was adapted from the Personal Science Teaching Efficacy Belief subscale of the Science Teaching Efficacy Belief Instrument Form B (STEBI-B), which was originally developed to measure pre-service teachers’ science teaching efficacy beliefs (Enochs and Riggs, 1990). Only the personal science teaching efficacy dimension was used because the present study focused on pre-service science teachers’ beliefs about their own teaching capability rather than science teaching outcome expectancy (Enochs and Riggs, 1990; Ingram et al., 2024). Seven adapted items assessed participants’ confidence in teaching scientific concepts, organizing science learning activities, supporting students’ scientific understanding, and managing inquiry-based science teaching.

#### 3.2.4 Intention to integrate AI into inquiry-based science teaching

The scale measuring intention to integrate AI into inquiry-based science teaching was adapted from items used by Ramnarain et al. (2025) to assess pre-service science teachers’ intention to use generative AI in inquiry-based teaching. Scale development was also informed by recent research on pre-service science teachers’ preparedness for AI integration and perceptions of generative AI in science education (Amandoron et al., 2025; Ishmuradova et al., 2025). Six adapted items assessed participants’ willingness to use AI in future science classrooms to support inquiry-question design, simulations, data interpretation, scientific modeling, experimental activities, and the construction of scientific explanations.

### 3.3 Translation and adaptation

Because all participants were Chinese pre-service science teachers, the original English-language scales were translated into Chinese and adapted to the context of AI-supported inquiry-based science teaching. A translation and back-translation procedure was used to support linguistic accuracy, semantic equivalence, and contextual appropriateness. First, two bilingual researchers independently translated the original English items into Chinese. A third researcher then compared and integrated the two Chinese versions, resolved wording discrepancies, and produced a preliminary Chinese questionnaire. During this process, the research team sought to preserve the core meaning of the original items while appropriately adapting selected expressions to Chinese pre-service science teachers’ learning experiences and prospective science teaching contexts.

Next, a bilingual expert who had not reviewed the original English scales back-translated the Chinese questionnaire into English. The research team compared the back-translated version with the original instruments to identify possible discrepancies in meaning, tone, and construct representation. Items with semantic inconsistencies or ambiguous contextual wording were revised through discussion. After back-translation, three experts in science education, educational technology, and teacher education reviewed the adapted Chinese items. They evaluated each item for content relevance, clarity, cultural appropriateness, and alignment with the intended construct. All item-level content validity index (I-CVI) values exceeded 0.78, and the scale-level content validity index (S-CVI) exceeded 0.90. The expert review therefore provided evidence of satisfactory content validity. Most items were rated as relevant or highly relevant to their intended constructs, and both item- and scale-level indices met recommended criteria. Based on the experts’ suggestions, minor wording changes were made to several items, particularly those concerning AI applications, scientific inquiry, and ethical evaluation, to improve clarity and contextual relevance.

Before the formal survey, a pilot test was conducted with 30 Chinese pre-service science teachers who were not included in the final sample. After completing the questionnaire, pilot participants provided feedback on item clarity, ambiguous wording, response difficulty, and completion time. The questionnaire was generally considered clear and understandable, and participants reported that the items were relevant to their learning experiences, AI use, and prospective science teaching contexts. The average completion time was acceptable, indicating that the questionnaire length was appropriate. Some participants considered several items concerning AI tools and inquiry-based science teaching to be overly abstract. These items were therefore revised to include more concrete expressions, such as visualization, simulation, data interpretation, and scientific explanation. Preliminary reliability analysis of the pilot data indicated acceptable internal consistency for all four constructs, with Cronbach’s alpha values above commonly accepted thresholds. The revised questionnaire was consequently considered suitable for the formal survey.

### 3.4 Data analysis

The absolute skewness and kurtosis values of the construct scores were below 0.22, indicating no serious univariate nonnormality.

The choice of PLS-SEM was based primarily on the study objectives rather than sample size or distributional characteristics. Compared with covariance-based SEM, which emphasizes covariance matrix reproduction, overall model fit, and confirmation of mature theories, PLS-SEM is especially appropriate when the research focus is on explaining variance in key endogenous constructs and examining predictive associations (Hair et al., 2019). This study aimed to explain variation in pre-service science teachers’ intention to integrate AI into inquiry-based science teaching and to test multiple direct and specific indirect associations among AI literacy, AI-TPACK, and science teaching self-efficacy. Because the model included emerging AI-related constructs adapted to a specific context and emphasized explained variance and serial indirect associations, PLS-SEM was aligned with the analytical objectives. Before the main analysis, missing data, invalid responses, outliers, and distributional characteristics were examined, followed by descriptive statistics. The reflective measurement model was then evaluated using outer loadings, Cronbach’s α, ρ_A, composite reliability (ρ_C), average variance extracted (AVE), and the heterotrait-monotrait ratio (HTMT). After the measurement model met the recommended criteria, the structural model was assessed using inner VIF values, path coefficients, R^2^, adjusted R^2^, f^2^, and Stone-Geisser Q^2^. The significance of direct and indirect associations was tested using 5,000 bias-corrected bootstrap samples. An indirect association was considered statistically significant when its 95% confidence interval did not include zero.

## 4 Data analysis and results

### 4.1 Descriptive analysis

As shown in Table 2, the construct means ranged from 4.68 to 5.15, indicating generally positive self-perceptions. Science teaching self-efficacy had the highest mean, whereas intention to integrate AI into inquiry-based science teaching had the lowest. Thus, although the pre-service science teachers reported relatively high levels of science teaching capability and AI-related pedagogical knowledge, their intention to use AI in future inquiry-based science teaching was comparatively lower. All skewness and kurtosis values were within acceptable ranges, indicating no serious univariate nonnormality. Because all focal variables were measured through the same self-report questionnaire, Harman’s single-factor test and full-collinearity VIFs were used to diagnose potential common method bias. First, all 30 measurement items were entered into an unrotated principal component analysis. Four components with eigenvalues greater than 1 were extracted, and the first component explained 44.71% of the total variance, below the commonly used 50% criterion. Second, following Kock’s (2015) full-collinearity assessment approach, each construct was successively treated as the dependent variable and predicted by the remaining constructs. The full-collinearity VIFs for AI literacy, AI-TPACK, science teaching self-efficacy, and AI integration intention were 1.592, 1.864, 1.785, and 1.547, respectively, all below 3.30. Taken together, these diagnostics did not indicate that common method bias was likely to pose a serious threat to the findings.

**Table 2:** Descriptive statistics.

Construct	M	SD	Skewness	Kurtosis
AIL	4.89	0.89	−0.07	−0.05
AITPACK	5.00	0.85	−0.16	−0.03
STSE	5.15	0.81	−0.07	−0.22
INTAI-IBST	4.68	0.95	−0.12	−0.14

### 4.2 Measurement model assessment

Table 3 presents the reliability and convergent validity results. Cronbach’s α values ranged from 0.920 to 0.948, ρ_A values ranged from 0.922 to 0.948, and composite reliability values ranged from 0.938 to 0.955, indicating strong internal consistency reliability for all constructs. AVE values ranged from 0.680 to 0.715 and exceeded the recommended threshold of 0.50, supporting convergent validity (Fornell and Larcker, 1981; Hair et al., 2021).

**Table 3:** Construct reliability and convergent validity.

Construct	Outer loading range	Cronbach’s α	Reliability (ρ_A)	Composite reliability (ρ_C)	Average variance extracted (AVE)
INTAI-IBST	0.783–0.856	0.920	0.922	0.938	0.715
AITPACK	0.796–0.844	0.948	0.948	0.955	0.680
AIL	0.811–0.843	0.923	0.924	0.938	0.683
STSE	0.819–0.867	0.924	0.924	0.939	0.686

Table 4 reports the HTMT assessment of discriminant validity. HTMT is a sensitive criterion for evaluating discriminant validity in PLS-SEM. Values below 0.85 generally indicate strong discriminant validity, whereas values below 0.90 are considered acceptable (Hair et al., 2019; Henseler et al., 2015). In this study, HTMT values ranged from 0.468 to 0.619 and were all below the more stringent 0.85 threshold, indicating adequate empirical distinctiveness among the latent constructs. In particular, the HTMT value between AI literacy and AI-TPACK was 0.609, showing that these conceptually related constructs remained empirically distinguishable.

**Table 4:** Heterotrait-monotrait ratio (HTMT).

Construct	INTAI-IBST	AITPACK	AIL	STSE
AITPACK	0.520			
AIL	0.468	0.609		
STSE	0.589	0.619	0.518	

### 4.3 Structural model assessment

Table 5 reports the inner VIF values assessed separately for the predictor constructs of each endogenous construct. Following Hair et al. (2019), a VIF below 5.00 was used as the primary criterion, while values below 3.00 indicate no notable collinearity among predictors. All inner VIF values ranged from 1.000 to 1.809, indicating no material collinearity problem in the structural model. The VIF for AI literacy predicting AI-TPACK was 1.000 because this endogenous construct had only one predictor, precluding collinearity among multiple predictors.

**Table 5:** Collinearity assessment among predictor constructs based on inner VIF values.

Predictor construct	AI-TPACK	STSE	INTAI-IBST
AI literacy	1.000	1.482	1.560
AI-TPACK	—	1.482	1.809
STSE	—	—	1.588

Table 6 reports the R^2^ and adjusted R^2^ values for the endogenous constructs. In PLS-SEM, R^2^ represents the proportion of variance in an endogenous construct explained by its predictors and is commonly used to assess the model’s explanatory power (Hair et al., 2019). The model explained 35.5% of the variance in intention to integrate AI into inquiry-based science teaching, 32.5% of the variance in AI-TPACK, and 37.0% of the variance in science teaching self-efficacy.

**Table 6:** R^2^ and adjusted R^2^ values.

Construct	R^2^	Adjusted R^2^
INTAI-IBST	0.355	0.351
AITPACK	0.325	0.324
STSE	0.370	0.368

Table 7 presents the f^2^ effect sizes. Following Cohen (1988) and Hair et al. (2019), values of 0.02, 0.15, and 0.35 can be interpreted as small, medium, and large effects, respectively. AI literacy had a large effect on AI-TPACK (f^2^ = 0.482), whereas AI-TPACK had a medium effect on science teaching self-efficacy (f^2^ = 0.221). For intention to integrate AI into inquiry-based science teaching, science teaching self-efficacy had the largest effect among the predictors, although it remained below the conventional medium-effect threshold (f^2^ = 0.127). The effects of AI-TPACK (f^2^ = 0.032) and AI literacy (f^2^ = 0.022) were small. These results indicate that science teaching self-efficacy contributed more to explaining AI integration intention than the direct contributions of AI literacy or AI-TPACK.

**Table 7:** F^2^ effect sizes.

Construct	INTAI-IBST	AITPACK	STSE
INTAI-IBST	—	—	—
AITPACK	0.032	—	0.221
AIL	0.022	0.482	0.053
STSE	0.127	—	—

Table 8 reports the Stone-Geisser Q^2^ values. In PLS-SEM, Q^2^ values greater than zero indicate in-sample predictive relevance for an endogenous construct (Geisser, 1974; Hair et al., 2019; Stone, 1974). The Q^2^ values for INTAI-IBST, AI-TPACK, and STSE were 0.250, 0.218, and 0.250, respectively, and were all above zero. The model therefore demonstrated in-sample predictive relevance for each endogenous construct.

**Table 8:** Stone–Geisser Q^2^ results.

Construct	SSO	SSE	Q^2^ (=1−SSE/SSO)
INTAI-IBST	3288.000	2464.724	0.250
AITPACK	5480.000	4283.317	0.218
STSE	3836.000	2877.322	0.250

### 4.4 Direct associations and hypothesis testing

**Figure 2:**
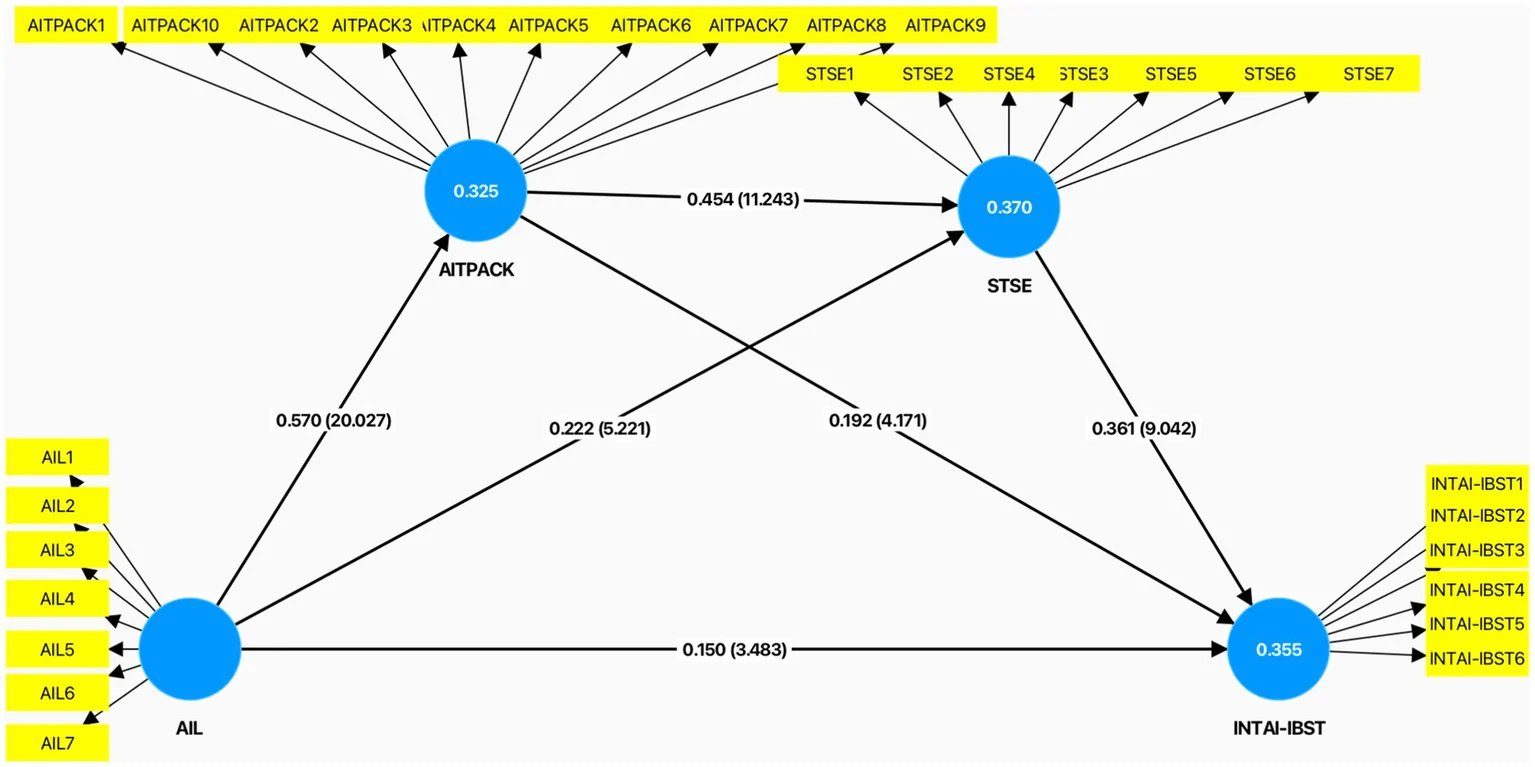
Research model and structural estimates.

Table 9 and Figure 2 show that all six direct paths were positive and statistically significant. AI literacy significantly predicted AI-TPACK (β = 0.570, 95% CI [0.513, 0.624]), supporting H1; AI-TPACK significantly predicted science teaching self-efficacy (β = 0.454, 95% CI [0.374, 0.533]), supporting H2; and science teaching self-efficacy significantly predicted AI integration intention (β = 0.361, 95% CI [0.282, 0.438]), supporting H3. The three additional direct paths retained for assessing mediation patterns were also significant.

**Table 9:** Structural path estimates.

Path	β	SE	*t*	*p*	95% CI	Result
AI literacy → AI-TPACK	0.570	0.028	20.027	<0.001	[0.513, 0.624]	Supported
AI-TPACK → STSE	0.454	0.040	11.243	<0.001	[0.374, 0.533]	Supported
STSE → INTAI-IBST	0.361	0.040	9.042	<0.001	[0.282, 0.438]	Supported
AI literacy → STSE	0.222	0.043	5.221	<0.001	[0.139, 0.307]	Significant
AI literacy → INTAI-IBST	0.150	0.043	3.483	0.001	[0.063, 0.231]	Significant
AI-TPACK→INTAI-IBST	0.192	0.046	4.171	<0.001	[0.100, 0.284]	Significant

### 4.5 Indirect associations and mediation patterns

The 95% confidence intervals for all five specific indirect associations excluded zero. As shown in Table 10, the indirect association between AI literacy and AI integration intention through AI-TPACK was significant (β = 0.110), supporting H4. The indirect association between AI-TPACK and AI integration intention through science teaching self-efficacy was significant (β = 0.164), supporting H5. The serial indirect association between AI literacy and AI integration intention through AI-TPACK and science teaching self-efficacy was also significant (β = 0.093), supporting H6. Because the direct paths from AI literacy and AI-TPACK to AI integration intention remained significant and had the same direction as the corresponding indirect associations, H4, H5, and H6 were interpreted as complementary partial mediation. The total indirect association between AI literacy and AI integration intention was 0.283 (95% CI [0.227, 0.344]), and the total association was 0.433 (95% CI [0.369, 0.496]). The total association between AI-TPACK and AI integration intention was 0.356 (95% CI [0.270, 0.443]).

**Table 10:** Specific indirect associations and mediation conclusions (5,000 bootstrap samples).

Indirect path	*β*	SE	*t*	*p*	95% CI	Interpretation
AI literacy → AI-TPACK → INTAI-IBST	0.110	0.028	3.949	<0.001	[0.057, 0.167]	Complementary partial mediation
AI literacy → AI-TPACK → STSE → INTAI-IBST	0.093	0.013	6.961	<0.001	[0.070, 0.121]	Complementary partial mediation
AI literacy → AI-TPACK → STSE	0.259	0.027	9.543	<0.001	[0.207, 0.314]	Significant indirect association
AI-TPACK → STSE → INTAI-IBST	0.164	0.022	7.404	<0.001	[0.123, 0.209]	Complementary partial mediation
AI literacy → STSE → INTAI-IBST	0.080	0.019	4.165	<0.001	[0.045, 0.121]	Significant indirect association

### 4.6 Robustness analysis

To assess whether the findings were sensitive to individual background characteristics, AI tool use frequency, AI-related training experience, and teaching practicum experience were added to the original set of controls. Gender, year of study, major, and university type were also controlled. Year of study, major, and AI use frequency were dummy coded, with second-year students, science education majors, and rare AI users as the respective reference groups. Men, normal-university students, participants without AI training, and participants without teaching practicum experience served as the reference groups for the other binary variables. All controls were entered simultaneously as predictors of AI-TPACK, science teaching self-efficacy (STSE), and intention to integrate AI into inquiry-based science teaching (INTAI-IBST).

A two-stage control analysis was conducted using standardized construct scores and 5,000 case-wise bootstrap samples. After the controls were added, the explained variance in AI-TPACK, STSE, and INTAI-IBST increased from 0.324, 0.369, and 0.353 to 0.333, 0.383, and 0.370, respectively. The incremental effect sizes were 0.012, 0.024, and 0.026, indicating that the controls provided only limited additional explanatory power.

As shown in Table 11, the direction and significance of every focal path remained unchanged after the controls were included, and the absolute changes in path coefficients did not exceed 0.008. Specifically, the predictive associations from AIL to AI-TPACK (β = 0.567, *p* < 0.001), AIL to STSE (β = 0.221, *p* < 0.001), AI-TPACK to STSE (β = 0.456, *p* < 0.001), AIL to INTAI-IBST (β = 0.156, *p* < 0.001), AI-TPACK to INTAI-IBST (β = 0.199, *p* < 0.001), and STSE to INTAI-IBST (β = 0.352, *p* < 0.001) all remained significant.

**Table 11:** Comparison of structural paths in the baseline and control models.

Structural path	Baseline β	Controlled β	Δβ	95% bootstrap CI	*p*
AIL → AI-TPACK	0.570	0.567	−0.003	[0.506, 0.624]	<0.001
AIL → STSE	0.222	0.221	−0.001	[0.136, 0.306]	<0.001
AI-TPACK → STSE	0.454	0.456	0.002	[0.374, 0.534]	<0.001
AIL → INTAI-IBST	0.150	0.156	0.006	[0.068, 0.241]	<0.001
AI-TPACK → INTAI-IBST	0.192	0.199	0.007	[0.107, 0.290]	<0.001
STSE → INTAI-IBST	0.361	0.352	−0.008	[0.270, 0.431]	<0.001

β values are standardized coefficients; Δβ equals the coefficient with controls minus the baseline coefficient. Bootstrap resamples = 5,000.

None of the controls significantly predicted INTAI-IBST directly, including AI use frequency, AI training experience, teaching practicum experience, year of study, major, gender, and university type. AI use frequency showed limited associations with STSE: compared with participants who rarely used AI, those who used AI sometimes (β = 0.099, *p* = 0.032) or often (β = 0.096, *p* = 0.033) reported higher STSE, whereas very frequent AI use was not significant. Gender also showed a weak association with AI-TPACK, with women reporting comparatively lower AI-TPACK. After the controls were included, the serial indirect association linking AIL with INTAI-IBST through AI-TPACK and STSE remained significant (β = 0.091, 95% CI [0.065, 0.120], *p* < 0.001). Thus, the focal structural associations and serial mediation findings were not materially altered by the background variables, supporting the robustness of the results.

## 5 Discussion

This study examined the mechanism linking AI literacy with Chinese pre-service science teachers’ intention to integrate AI into inquiry-based science teaching. The results supported the proposed structural model: AI literacy significantly predicted AI-TPACK, AI-TPACK significantly predicted science teaching self-efficacy, and science teaching self-efficacy significantly predicted intention to integrate AI into inquiry-based science teaching. These findings indicate that pre-service science teachers’ future intention to use AI in science classrooms is not associated with AI literacy alone. It is also embedded in a broader professional learning process involving AI-integrated pedagogical knowledge and confidence in science teaching.

First, the significant association between AI literacy and AI-TPACK indicates that pre-service science teachers with stronger AI literacy were more likely to perceive themselves as capable of integrating AI with science content and pedagogy. This finding is consistent with evidence that GenAI can support science lesson planning, scientific explanation, visualization, and inquiry-based instructional design (Lee and Zhai, 2024). It also extends Antonio's (2025) intervention-based evidence that AI-focused pedagogical learning can enhance pre-service science teachers’ AI-TPACK. In the present study, AI literacy appeared to provide a foundation for AI-TPACK because teachers need to understand, evaluate, and ethically use AI tools before applying them to scientific modeling, experimental simulation, data interpretation, and inquiry-activity design. From the perspectives of DPACK and DiKoLAN AI, however, the AI-TPACK measured in this study primarily reflects participants’ perceived AI-integrated pedagogical knowledge. It did not directly encompass the sociocultural consequences of digitality or actual competence in data processing, simulation and modeling, and scientific information evaluation. AI-TPACK is therefore better understood here as a professional knowledge bridge between general AI literacy and AI integration intention rather than as a comprehensive representation of science teachers’ AI competence. Maurer et al. (2026) found no significant association between general AI literacy and science educators’ actual AI use, whereas the present study examined pre-service teachers’ self-reported knowledge and future use intention. The two findings are therefore not contradictory. Together, they suggest that general AI literacy may become associated with teaching confidence and integration intention only when it is further contextualized through AI-TPACK that connects AI with science content and pedagogy. AI literacy is consequently a necessary foundation but not a sufficient condition for science teachers’ AI-related professional competence.

Second, the positive association between AI-TPACK and science teaching self-efficacy suggests that AI-TPACK is not merely a form of technological competence but also an efficacy-relevant knowledge base. When pre-service science teachers understand how to align AI tools with science content and inquiry-based pedagogy, they may perceive themselves as more capable of designing, managing, and improving science learning activities. This interpretation is consistent with the intelligent-TPACK perspective, which emphasizes that AI integration requires teachers to connect technological, pedagogical, content, and ethical knowledge (Çelik, 2023). It also accords with evidence that structured, technology-enhanced, and student-centered learning experiences can strengthen pre-service teachers’ personal science teaching efficacy (Ingram et al., 2024; Ribeirinha and Correia, 2025). AI-TPACK may therefore function as a cognitive source of science teaching self-efficacy in AI-supported science teacher education.

Third, science teaching self-efficacy significantly predicted pre-service science teachers’ intention to integrate AI into inquiry-based science teaching. This finding indicates that pre-service teachers may not intend to use AI merely because they understand AI tools or recognize their usefulness. Rather, intention appears stronger when they believe that they can use AI effectively to support students’ scientific understanding, inquiry processes, data interpretation, and explanation construction. This result extends Ramnarain et al. (2025), who identified several predictors of pre-service science teachers’ intention to use generative AI in inquiry-based teaching, by highlighting science teaching self-efficacy as an important psychological mechanism associated with AI integration intention. It is also consistent with recent evidence that purposeful teacher education experiences can strengthen pre-service teachers’ science teaching efficacy (Ingram et al., 2024; Ribeirinha and Correia, 2025).

Finally, the results statistically supported the proposed professional learning sequence from AI literacy to AI-TPACK, science teaching self-efficacy, and AI integration intention. The serial indirect association indicates that AI literacy was associated with AI integration intention both directly and indirectly through AI-integrated pedagogical knowledge and confidence in science teaching. This finding helps explain why general AI literacy alone may be insufficient to prepare pre-service science teachers for AI-supported science education. To connect AI literacy with future teaching intention, teacher education programs need to support the development of AI-TPACK and confidence in using AI for inquiry-based science teaching. The study therefore extends intention-centered research by shifting attention from whether pre-service teachers intend to use AI to how AI literacy is associated with teaching intention through professional knowledge and self-efficacy.

## 6 Contributions and future research directions

### 6.1 Theoretical contributions

This study makes three theoretical contributions. First, it extends research on AI in teacher education by shifting attention from general AI acceptance to the professional learning mechanisms associated with AI-integrated science teaching. Previous studies have examined pre-service science teachers’ intention to use generative AI in inquiry-based teaching and identified predictors including AI literacy, perceived usefulness, attitudes, subjective norms, and perceived behavioral control (Ramnarain et al., 2025). The present study goes beyond identifying predictors of AI use intention by explaining how AI literacy is associated with AI-TPACK, science teaching self-efficacy, and AI integration intention. It therefore responds to an emerging need in AI teacher education to move from tool adoption toward subject-specific pedagogical integration mechanisms.

Second, by situating AI-TPACK within the theoretical development of TPACK, DPACK, and DiKoLAN AI, this study clarifies both the role and the boundaries of AI-TPACK in pre-service science teacher education. The results suggest that perceived AI-TPACK may constitute a professional knowledge bridge between general AI literacy and science teaching self-efficacy. This finding supports TPACK’s core proposition that technological, pedagogical, and content knowledge must be integrated contextually, while responding to the need for teachers in the AI era to coordinate technological knowledge, content judgment, instructional design, and ethical responsibility. At the same time, the study does not treat AI-TPACK as a comprehensive framework encompassing all teacher AI competence. Its theoretical contribution is not to demonstrate that AI-TPACK is superior to other frameworks, but to position it as a proximal professional knowledge construct linking general AI literacy, science teaching confidence, and AI integration intention, while clarifying its relationship with broader sociocultural knowledge and subject-specific practice.

Third, this study contributes to teacher self-efficacy research by indicating that AI-supported pedagogical knowledge may constitute a cognitive source of science teaching self-efficacy in the AI era. Recent studies show that structured, technology-enhanced, and practice-oriented learning experiences can strengthen pre-service teachers’ science teaching efficacy beliefs (Ingram et al., 2024; Ribeirinha and Correia, 2025). Building on this evidence, the present study suggests that AI-TPACK may serve as a knowledge base associated with self-efficacy. In AI-supported science teacher education, self-efficacy may therefore be informed not only by direct teaching practice but also by the construction of subject-specific AI-related pedagogical knowledge.

### 6.2 Practical implications

The findings have several implications for science teacher education. First, teacher educators should not limit AI training to general tool operation, such as using ChatGPT or generating teaching materials. AI training should instead be embedded within science pedagogy and inquiry-based teaching. Teacher educators can guide pre-service science teachers in using AI to design inquiry questions, evaluate AI-generated scientific models, employ simulations to support experimental reasoning, judge the scientific accuracy of AI-generated content, and identify ethical risks and biases in AI-supported instruction. This implication is consistent with recent AI-TPACK research indicating that AI integration requires instructional design, ethical reflection, and subject-specific application rather than isolated technical skills training (Antonio, 2025; Çelik, 2023).

Second, universities should incorporate AI-TPACK into science teacher education curricula rather than addressing AI only in general educational technology courses. Science education programs could offer specialized modules that guide pre-service teachers in using AI for scientific concept visualization, experimental simulation, data interpretation, scientific explanation construction, and inquiry-based lesson design. Such curricular design may help pre-service science teachers contextualize general AI literacy as subject-specific AI-related pedagogical knowledge. This approach is also consistent with the AI competency framework for teachers, which identifies AI ethics, AI foundations and applications, AI pedagogy, and AI-supported professional learning as important dimensions of teacher competence in the AI era (Miao and Cukurova, 2024).

Third, the findings have implications for policymakers, particularly as science education reform and AI-enabled educational reform advance simultaneously. Recent Chinese policy documents emphasize AI-supported educational reform, digital transformation, and the development of AI literacy systems across all levels of education (General Office of the Ministry of Education of the People’s Republic of China, 2025; Ministry of Education of the People’s Republic of China et al., 2026). Policy implementation should therefore extend beyond general AI courses or digital literacy training to include assessment tools and curricula that address pre-service science teachers’ capacity to use AI in science teaching. Teacher education systems should evaluate not only whether pre-service teachers possess AI knowledge, but also whether they can apply AI to scientific inquiry, model-based reasoning, experimental activities, data interpretation, and ethical classroom practice.

### 6.3 Limitations and future research

This study has several limitations. First, its cross-sectional design precludes strong causal inference. Although the serial mediation model was theoretically grounded and statistically supported, the findings should be interpreted as associations rather than a demonstrated developmental sequence. Future research should use longitudinal or experimental designs to examine how AI literacy, AI-TPACK, science teaching self-efficacy, and AI integration intention change over time.

Second, the data were collected through a self-report questionnaire and may therefore be affected by social desirability or participants’ subjective perceptions. Future studies could combine AI-supported instructional design tasks, AI use logs, classroom observations, microteaching performance, or expert ratings to provide more objective evidence.

Third, the sample comprised Chinese pre-service science teachers. The extent to which the findings generalize to other countries, regions, or types of teacher education institutions requires further investigation.

Fourth, science teacher education encompasses physics, chemistry, biology, earth science, and integrated science, and AI integration may vary according to disciplinary content and teaching tasks. Future research could therefore conduct multigroup comparisons to examine the applicability of the model across science disciplines. Science teacher education should also move beyond general AI tool operation and ethical knowledge by embedding AI in disciplinary practices such as data analysis, simulation and modeling, scientific explanation, evidence evaluation, and inquiry-activity design. Such an approach may help contextualize general AI literacy as subject-specific AI-related pedagogical knowledge.

Fifth, this study used AI-TPACK as the primary construct representing AI-integrated pedagogical knowledge. It did not directly measure the sociocultural knowledge emphasized by DPACK or use performance tasks to assess the subject-specific competencies identified in DiKoLAN AI, such as data processing, simulation and modeling, scientific information evaluation, and AI-supported feedback. The results therefore primarily reflect pre-service science teachers’ subjective perceptions of AI-integrated pedagogical knowledge. Future research could integrate AI-TPACK, DPACK, and DiKoLAN AI and combine self-reports with instructional design tasks, classroom observations, and performance-based assessments to develop a more comprehensive model of science teachers’ AI competence.

## 7 Conclusion

This study examined how AI literacy was associated with Chinese pre-service science teachers’ intention to integrate AI into inquiry-based science teaching through AI-TPACK and science teaching self-efficacy. The results supported the proposed serial mediation model: stronger AI literacy was associated with stronger AI-TPACK, which was in turn associated with greater science teaching self-efficacy and, ultimately, stronger AI integration intention. These findings indicate that preparing pre-service science teachers for AI-supported science education requires more than strengthening general AI literacy. Teacher education programs should help pre-service science teachers contextualize AI literacy as subject-specific AI-related pedagogical knowledge and build confidence in science teaching. By highlighting the sequential roles of AI-TPACK and science teaching self-efficacy, this study provides empirical support for theory-informed teacher education practices intended to prepare pre-service science teachers to use AI in inquiry-based science teaching. The outcome examined here is teaching intention, which reflects psychological readiness rather than demonstrated AI integration competence or classroom performance.

## Data Availability

The datasets presented in this article are not readily available because due to privacy and ethical restrictions, the raw datasets generated and analyzed during the current study are not publicly available. Anonymized data may be made available from the corresponding author upon reasonable request and subject to appropriate ethical and confidentiality considerations. Requests to access the datasets should be directed to duj8180@gmail.com.
