# Supplementary material for: From AI literacy to AI-integrated inquiry-based science teaching: the serial mediating roles of AI-TPACK and science teaching self-efficacy among Chinese pre-service science teachers
Source: Front Psychol. 2026 Sep 7;17:1911909. doi: 10.3389/fpsyg.2026.1911909 (PMC13590729; doi:10.3389/fpsyg.2026.1911909)
Supplement: Supplementary file 1 [file Supplementary_File_1.docx]

**Questionnaire on AI-Supported Inquiry-Based Science Teachingfor Pre-Service Science Teachers**

# **Participant Information**

Dear Participant,
This questionnaire aims to understand Chinese pre-service science teachers' views of artificial intelligence (AI) tools and their application in inquiry-based science teaching. The questionnaire is anonymous and does not collect information that can directly identify you. Your participation is entirely voluntary, and you may stop at any time before submitting your responses. All data will be used solely for academic research and will be analyzed and reported only in aggregate. There are no right or wrong answers. Please respond based on your actual experience and views.

**AI tools in this questionnaire:** Artificial intelligence systems capable of generating, processing, or analyzing text, images, charts, scientific data, simulations, audio, or video.

**□ I have read and understood the information above and voluntarily agree to participate in this study.**
□ I do not agree to participate and will stop completing this questionnaire.

# **Part I. Background Information**

Please check (✓) the option that best describes you.

| **Item** | **Select the option that best describes you** |
| --- | --- |
| **1. Gender** | □ Male □ Female |
| **2. Age** | □ 18-20 □ 21-22 □ 23 or older |
| **3. Year of study** | □ Second year □ Third year □ Fourth year |
| **4. Major/specialization** | □ Science Education □ Physics Education □ Chemistry Education □ Biology Education □ Geography Education |
| **5. Institution type** | □ Teacher-training university □ Comprehensive university |
| **6. Teaching practicum experience** | □ Yes □ No |
| **7. Relevant coursework** | □ Educational technology or science teaching methods: Yes □ No |
| **8. Frequency of AI tool use** | □ Rarely □ Sometimes □ Often □ Very often |
| **9. AI-related training experience** | □ Yes □ No |

# **Part II. Core Measurement Items**

Please indicate your level of agreement with each statement by checking (✓) one number. Select only one response for each item.

**1 = Strongly disagree	2 = Disagree	3 = Somewhat disagree	4 = Neither agree nor disagree
5 = Somewhat agree	6 = Agree	7 = Strongly agree**

## **A. AI Literacy (AIL)**

These items assess your ability to understand, use, evaluate, and responsibly apply AI tools across different modalities.

| **Code** | **Statement** | **1** | **2** | **3** | **4** | **5** | **6** | **7** |
| --- | --- | --- | --- | --- | --- | --- | --- | --- |
| **AIL1** | I can identify AI tools that generate or analyze different types of content, including text, images, data, simulations, audio, and video. | □ | □ | □ | □ | □ | □ | □ |
| **AIL2** | I understand the basic functions and appropriate uses of different types of AI tools, as well as their limitations in science education. | □ | □ | □ | □ | □ | □ | □ |
| **AIL3** | I can use AI to generate or analyze scientific text, such as scientific explanations, inquiry questions, and content summaries. | □ | □ | □ | □ | □ | □ | □ |
| **AIL4** | I can use AI to generate or analyze scientific images, charts, models, and simulations. | □ | □ | □ | □ | □ | □ | □ |
| **AIL5** | I can use AI to process scientific data and identify patterns, trends, or relationships in the data. | □ | □ | □ | □ | □ | □ | □ |
| **AIL6** | I can use AI to generate or analyze audio and video materials related to science teaching. | □ | □ | □ | □ | □ | □ | □ |
| **AIL7** | I can evaluate the scientific accuracy, potential bias, and pedagogical appropriateness of multimodal AI outputs, while protecting privacy, respecting copyright, and disclosing the use of AI. | □ | □ | □ | □ | □ | □ | □ |

## **B. AI-TPACK**

These items assess your perceived ability to integrate AI tools, science content, and inquiry-based teaching approaches.

| **Code** | **Statement** | **1** | **2** | **3** | **4** | **5** | **6** | **7** |
| --- | --- | --- | --- | --- | --- | --- | --- | --- |
| **AITPACK1** | I can select appropriate AI tools based on the science content, inquiry goals, and student characteristics. | □ | □ | □ | □ | □ | □ | □ |
| **AITPACK2** | I can use AI-generated images, models, or simulations to help students understand abstract scientific concepts. | □ | □ | □ | □ | □ | □ | □ |
| **AITPACK3** | I can integrate AI into the process of formulating inquiry questions and scientific hypotheses. | □ | □ | □ | □ | □ | □ | □ |
| **AITPACK4** | I can appropriately integrate AI into the design and implementation of science experiments and related safety guidance. | □ | □ | □ | □ | □ | □ | □ |
| **AITPACK5** | I can use AI to support students in collecting, analyzing, and interpreting scientific data. | □ | □ | □ | □ | □ | □ | □ |
| **AITPACK6** | I can use AI to support students in constructing scientific models and evidence-based scientific explanations. | □ | □ | □ | □ | □ | □ | □ |
| **AITPACK7** | I can use AI to monitor students' inquiry processes and provide appropriate feedback and assessment. | □ | □ | □ | □ | □ | □ | □ |
| **AITPACK8** | I can design complete lessons that integrate AI tools, science content, and inquiry-based teaching approaches. | □ | □ | □ | □ | □ | □ | □ |
| **AITPACK9** | I can identify and correct scientific errors in AI-generated content to prevent misconceptions among students. | □ | □ | □ | □ | □ | □ | □ |
| **AITPACK10** | I can address ethical issues in AI-supported science teaching, including fairness, privacy, copyright, transparency, and differences among students. | □ | □ | □ | □ | □ | □ | □ |

## **C. Science Teaching Self-Efficacy (STSE)**

These items assess your confidence in your ability to implement AI-supported inquiry-based science teaching.

| **Code** | **Statement** | **1** | **2** | **3** | **4** | **5** | **6** | **7** |
| --- | --- | --- | --- | --- | --- | --- | --- | --- |
| **STSE1** | In the context of AI-supported inquiry-based science teaching, I am confident that I can explain scientific concepts and principles to students clearly and accurately. | □ | □ | □ | □ | □ | □ | □ |
| **STSE2** | In the context of AI-supported inquiry-based science teaching, I am confident that I can answer students' science questions and correct their misconceptions. | □ | □ | □ | □ | □ | □ | □ |
| **STSE3** | In the context of AI-supported inquiry-based science teaching, I am confident that I can design inquiry-based learning activities aligned with science learning objectives. | □ | □ | □ | □ | □ | □ | □ |
| **STSE4** | In the context of AI-supported inquiry-based science teaching, I am confident that I can effectively organize and manage the processes through which students formulate questions, develop hypotheses, and collect evidence. | □ | □ | □ | □ | □ | □ | □ |
| **STSE5** | In the context of AI-supported inquiry-based science teaching, I am confident that I can guide students in designing and conducting science experiments safely and effectively. | □ | □ | □ | □ | □ | □ | □ |
| **STSE6** | In the context of AI-supported inquiry-based science teaching, I am confident that I can guide students in analyzing data and drawing scientific conclusions or constructing explanations based on evidence. | □ | □ | □ | □ | □ | □ | □ |
| **STSE7** | In the context of AI-supported inquiry-based science teaching, I am confident that I can assess students' scientific understanding and adjust my teaching strategies based on their learning performance. | □ | □ | □ | □ | □ | □ | □ |

## **D. Intention to Integrate AI into Inquiry-Based Science Teaching (INTAI-IBST)**

These items assess your willingness and plans to use AI to support inquiry-based teaching in future science classrooms.

| **Code** | **Statement** | **1** | **2** | **3** | **4** | **5** | **6** | **7** |
| --- | --- | --- | --- | --- | --- | --- | --- | --- |
| **INTAI1** | When I become a science teacher, I plan to use AI tools in inquiry-based science teaching. | □ | □ | □ | □ | □ | □ | □ |
| **INTAI2** | When I become a science teacher, I am willing to use AI to help design activities involving inquiry questions and scientific hypothesis formulation. | □ | □ | □ | □ | □ | □ | □ |
| **INTAI3** | When I become a science teacher, I plan to use AI-generated visualizations, models, or simulations to support scientific inquiry. | □ | □ | □ | □ | □ | □ | □ |
| **INTAI4** | When I become a science teacher, I am willing to use AI to support the design and implementation of science experiments or the collection of evidence. | □ | □ | □ | □ | □ | □ | □ |
| **INTAI5** | When I become a science teacher, I plan to use AI to support students in analyzing scientific data and interpreting results. | □ | □ | □ | □ | □ | □ | □ |
| **INTAI6** | When I become a science teacher, I am willing to use AI to help students construct evidence-based scientific explanations. | □ | □ | □ | □ | □ | □ | □ |

**This concludes the questionnaire. Thank you sincerely for your participation.**

Please ensure that you have answered all items before submitting the questionnaire.
